# Prevalence and risk factors of carbapenem-resistant *Enterobacterales* positivity by active screening in intensive care units in the Henan Province of China: A multi-center cross-sectional study

**DOI:** 10.3389/fmicb.2022.894341

**Published:** 2022-09-14

**Authors:** Bo Guo, Ziqi Guo, Huifeng Zhang, Chuanchuan Shi, Bingyu Qin, Shanmei Wang, Yinjiang Chang, Jian Chen, Peili Chen, Limin Guo, Weidong Guo, Huaibin Han, Lihong Han, Yandong Hu, Xiaoye Jin, Yening Li, Hong Liu, Ping Lou, Yibing Lu, Panfeng Ma, Yanhua Shan, Yiyi Sun, Wukui Zhang, Xisheng Zheng, Huanzhang Shao

**Affiliations:** ^1^Department of Critical Care Medicine, Henan Provincial People’s Hospital, Zhengzhou, China; ^2^Department of Critical Care Medicine, Henan University People’s Hospital, Zhengzhou, China; ^3^Henan Key Laboratory for Critical Care Medicine, Zhengzhou, China; ^4^Department of Critical Care Medicine, Zhengzhou University People’s Hospital, Zhengzhou, China; ^5^Department of Microbiology Laboratory, Henan Provincial People’s Hospital, Zhengzhou, China; ^6^Department of Critical Care Medicine, Puyang People’s Hospital, Puyang, China; ^7^Department of Critical Care Medicine, Xuchang Central Hospital, Xuchang, China; ^8^Department of Critical Care Medicine, Shangqiu People’s Hospital, Shangqiu, China; ^9^Department of Critical Care Medicine, Jiyuan People’s Hospital, Jiyuan, China; ^10^Department of Critical Care Medicine, Xinxiang Central Hospital, Xinxiang, China; ^11^Department of Critical Care Medicine, The Fourth Clinical College of Xinxiang Medical College, Xinxiang, China; ^12^Department of Critical Care Medicine, Zhoukou Central Hospital, Zhoukou, China; ^13^Department of Critical Care Medicine, Luoyang Central Hospital, Luoyang, China; ^14^Department of Critical Care Medicine, Sanmenxia Central Hospital, Sanmenxia, China; ^15^Department of Critical Care Medicine, Kaifeng People’s Hospital, Kaifeng, China; ^16^Department of Critical Care Medicine, Luohe Central Hospital, Luohe, China; ^17^Department of Critical Care Medicine, Pingdingshan First People’s Hospital, Pingdingshan, China; ^18^Department of Critical Care Medicine, Zhengzhou First People’s Hospital, Zhengzhou, China; ^19^Department of Critical Care Medicine, Xinyang Central Hospital, Xinyang, China; ^20^Department of Critical Care Medicine, Anyang People’s Hospital, Anyang, China; ^21^Department of Critical Care Medicine, Zhumadian Central Hospital, Zhumadian, China; ^22^Department of Critical Care Medicine, Hebi People’s Hospital, Hebi, China; ^23^Department of Critical Care Medicine, Jiaozuo People’s Hospital, Jiaozuo, China; ^24^Department of Critical Care Medicine, Nanyang Central Hospital, Nanyang, China

**Keywords:** carbapenem-resistant *Enterobacterales* (CRE), prevalence, intensive care unit, bacterial resistance, active screening

## Abstract

**Objective:**

In intensive care units (ICUs), carbapenem-resistant *Enterobacterales* (CRE) pose a significant threat. We aimed to examine the distribution, epidemiological characteristics, and risk factors for CRE positivity in ICUs.

**Materials and methods:**

This cross-sectional study was conducted in 96 ICUs of 78 hospitals in Henan Province, China. The clinical and microbiological data were collected. A multivariable logistic regression model was used to analyze the risk factors for CRE positivity.

**Results:**

A total of 1,009 patients were enrolled. There was a significant difference in CRE positive rate between pharyngeal and anal swabs (15.16 vs. 19.13%, *P* < 0.001). A total of 297 carbapenem-resistant *Klebsiella pneumoniae* (CR-KPN), 22 carbapenem-resistant *Escherichia coli* (CR-ECO), 6 carbapenem-resistant *Enterobacter cloacae* (CR-ECL), 19 CR-KPN/CR-ECO, and 2 CR-KPN/CR-ECL were detected. *Klebsiella pneumoniae* carbapenemase (KPC), New Delhi metallo-beta-lactamase (NDM), and a combination of KPC and NDM were detected in 150, 9, and 11 swab samples, respectively. Multivariable logistic regression analysis determined length of ICU stay, chronic neurological disease, transfer from other hospitals, previous infection, and history of antibiotics exposure as independent risk factors for CRE positivity. Age and cardiovascular diseases were independent risk factors for mixed infections of CRE. The occurrence of CRE in secondary and tertiary hospitals was 15.06 and 25.62%, respectively (*P* < 0.05). Patients from tertiary hospitals had different clinical features compared with those from secondary hospitals, including longer hospital stays, a higher rate of patients transferred from other hospitals, receiving renal replacement therapy, exposure to immunosuppressive drugs, use of antibiotics, and a higher rate of the previous infection.

**Conclusion:**

In ICUs in Henan Province, CRE positive rate was very high, mostly KPC-type CR-KPN. Patients with prolonged ICU stay, chronic neurological disease, transfer from other hospitals, previous infection, and history of antibiotic exposure are prone to CRE. Age and cardiovascular diseases are susceptibility factors for mixed infections of CRE. The CRE positive rate in tertiary hospitals was higher than that in secondary hospitals, which may be related to the source of patients, antibiotic exposure, disease severity, and previous infection.

## Introduction

Bacterial drug resistance is becoming a global public health concern. The prevalence of carbapenem-resistant Gram-negative bacteria is rising worldwide, posing a major threat to public health (Tängdén and Giske, 2015). China’s Antimicrobial Surveillance Network (CHINET) reported in 2020 that carbapenem-resistant *Klebsiella pneumoniae* (CR-KPN) prevalence increased slowly in China. Among these, the drug resistance rate of CR-KPN in Henan Province ranked top (Quan, 2020).

There is a significant morbidity and mortality associated with carbapenem-resistant *Enterobacterales* (CRE) infection (van Duin and Doi, 2017). According to a previous study, patients with sepsis caused by CRE had a significantly higher 30-day mortality than those with other infections (Sabino et al., 2019). According to another study, the treatment costs of CRE infection are higher than the annual costs of chronic diseases such as diabetes, asthma, and hypertension, resulting in a severe socioeconomic burden (Bartsch et al., 2017).

The presence of CRE colonization is a risk factor for CRE infection (Shu et al., 2020), which may increase the prevalence of CRE infection (Lin et al., 2021; Yin et al., 2021; Gomides et al., 2022). In addition, CRE colonization or infection was associated with longer hospital stays and an overall mortality of 10% (Tischendorf et al., 2016). Moreover, patients admitted to ICUs with CRE colonization had an increased risk of infections and mortality (McConville et al., 2017). The treatment options for CRE infection are limited, including polymyxins, tigecycline, aminoglycosides, and carbapenems (Iovleva and Doi, 2017). There is a high mortality rate due to the limited treatment options. Therefore, early detection and prevention of CRE (especially carbapenemase-producing CRE) are crucial to managing critically ill patients.

The World Health Organization (WHO), the US Centers for Disease Control (CDC), and the European CDC all recommend screening for CRE among patients at high risk for CRE colonization (Centers for Disease Control and Prevention, 2015; Magiorakos et al., 2017; World Health Organization, 2017). Since the ICU has a high incidence of CRE infection, CRE screening at the time of ICU admission and examining the CRE colonization status of patients are beneficial for early detection, and early isolation of CRE cases, which could aid to control the ICU-related CRE dissemination (Chen et al., 2017). Diagnosis and treatment costs can be reduced by detecting intestinal colonization of CRE rapidly and accurately (Berry et al., 2019).

This study aimed to perform CRE active screening in patients admitted to the ICU to determine the prevalence and risk factors for CRE positivity in Henan Province. The findings of the study would contribute to anti-infective therapy, infection prevention, and control of CRE.

## Materials and methods

### Patients

Patients over 18 years of age in ICUs were included in the study. All enrolled patients signed an informed consent form. This study involved 1,048 inpatients in 96 ICUs of 78 hospitals in 18 cities. Of these, 1,009 were study-eligible. Participants who met all of the study criteria were referred to the study if they adhered to the experimental protocol, which included the availability of variable measurements.

Among the 78 hospitals, 23 hospitals were in Zhengzhou city, six in Xuchang city, one in Anyang city, four in Puyang city, two in Zhoukou city, four in Jiaozuo city, five in Kaifeng city, six in Xinxiang city, one in Hebi city, three in Shangqiu city, two in Xinyang city, one in Jiyuan city, two in Luohe city, three in Zhumadian city, three in Sanmenxia city, three in Nanyang city, three in Luoyang city, and six in Pingdingshan city. Table 1 shows the distribution of hospitals.

**TABLE 1:** Levels of hospitals participated in the study.

	Classification	Hospitals (*n* = 78)
Hospital level	Provincial	16 (20.51%)
	Municipal	41 (52.56%)
	County-level	21 (26.92%)

### Study design and specimen collection

Swabs (one pharyngeal and two anal swabs per patient) from the enrolled patients were collected by the trained ICU nurses in participating hospitals on March 10, 2021. The collection of samples began at 8 a.m. and was completed on the same day. The samples were placed in sealed bags after being labeled. A dedicated individual was appointed to transport the specimens under environmental temperatures to Henan Provincial People’s Hospital’s microbiology laboratory within 12 h but no longer than 24 h for unified testing. One pharyngeal and anal swabs specimen were tested for microorganisms. For patients with CRE positivity, the other anal swab specimen was tested for carbapenemase type. Specimen collection and transport were performed following the recommendations of Specimen Collection and Transport in Clinical Microbiology (WS/T640-2018), and the People’s Republic of China Health Industry Standard. Each ICU had a doctor and a nurse assigned to collect specimens, complete case reports, follow-up with patients, and manage data.

The study was registered at http://www.chictr.org.cn/ (registration number: ChiCTR2100044002) before patient enrollment.

### Bacterial isolate collection and identification

All respiratory tract and stool specimens were enriched with selective fluids using a commercial kit (Xituo Biotechnology, China). First, the specimens were inoculated into a 3 ml conditioning solution and cultivated at 37°C for 6–8 h. Subsequently, 10 μl of fluid was extracted and subcultivated in 1 ml screening liquid, and meropenem screening supplements with a working concentration of 4 μg/ml were added, and the mixture was incubated at 37°C overnight. Finally, 10 μl of the reddened positive culture was taken and inoculated on a MacConkey agar plate medium and cultured in an incubator at 37°C for 24 h. Single colonies were isolated and identified by matrix-assisted laser desorption/ionization time-of-flight (MALDI-TOF) mass spectrometry (Bruker Daltonics). Meropenem-resistant strains were detected using the K-B disc diffusion method. Carbapenem-resistant *K. pneumoniae* (ATCC BAA-1705) and carbapenem-sensitive *K. pneumoniae* (ATCC BAA-1706) were the positive and negative control strains, respectively.

### Identification of carbapenemase types

All CRE anal swabs were tested for the most common carbapenemase types, including *Klebsiella pneumoniae* carbapenemase (KPC), New Delhi metallo-beta-lactamase (NDM), and oxacillinase-48 (OXA-48). The colloidal gold enzyme immunochromatography (NG test Carba 5) kit was purchased from Shanghai Fosun Pharmaceutical Co., Ltd. (Shanghai, China). The test was carried out in accordance with the manufacturer’s instructions. A total of 10 μl of bacteria was mixed with five drops of extraction buffer, and then 100 μl of the mixture was distributed into the CARBA-5 cassette. The results were interpreted after incubation at room temperature for 15 min.

### Statistical analysis

SPSS 26.0 was used for the statistical analysis, and multiple imputations was used to handle missing data (Li et al., 2015). The continuous data were presented as mean ± standard deviation or median and upper/lower quartiles according to normal distribution. For continuous data with a normal distribution and variance equality, paired samples *t*-tests were used, or for skewed distributions, Mann–Whitney *U*-tests were used. Categorical variables were compared using the Chi-square test. A multivariable logistic regression model was used to analyze the risk factors for CRE infection. All variables related to CRE positivity in the univariable analysis (*p* < 0.1) were included as candidate variables in the multivariable analysis, and stepwise regression was used to screen independent variables. Multicollinearity was performed before conducting the multivariate models, if the tolerance was greater than 0.1 and the variance inflation factor was less than 10, there was no multicollinearity among the variables. A *P*-value of < 0.05 was considered significant.

## Results

### Carbapenem-resistant *Enterobacterales* positive rates in different samples

This study included 1,009 pharyngeal swab specimens and 2,018 anal swab specimens collected from 1,009 hospitalized patients in 96 ICUs from 78 hospitals in Henan Province. A total of 346 CRE were isolated from the pharyngeal and anal swabs. The positive rates of CRE in the pharyngeal and anal swab samples were 15.16 and 19.13%, respectively, with a significant difference (*P* = 0.001) (Table 2).

**TABLE 2:** Positive rates in different types of samples.

Anal swabs	Pharyngeal swabs		Total	*P*-value
	Positive	Negative		
Positive	106	87	193 (19.13%)	0.001
Negative	47	769	816 (80.87%)	
Total	153 (15.16%)	856 (84.84%)	1009	

### Microbiological data in the carbapenem-resistant *Enterobacterales* positive specimens

A total of 297 carbapenem-resistant *Klebsiella pneumoniae* (CR-KPN) cases were detected in pharyngeal and anal swabs, including 264 and 33 cases from the tertiary and secondary hospitals, respectively. Carbapenem-resistant *Escherichia coli* (CR-ECO) (*n* = 22), carbapenem-resistant *Enterobacter cloacae* (CR-ECL) (*n* = 6), and CR-KPN/CR-ECL (mixed infections) (samples include two bacterial species: carbapenem-resistant *Klebsiella pneumoniae* and carbapenem-resistant *Enterobacter cloacae*) (*n* = 2) were all detected in the tertiary hospitals. This study detected 19 cases of CR-KPN/CR-ECO (mixed infections) (samples include two bacterial species: carbapenem-resistant *Klebsiella pneumoniae* and carbapenem-resistant *Escherichia coli*), 18 of which were detected in tertiary hospitals while one was detected in secondary hospitals (Table 3).

**TABLE 3:** Strains detected in different level hospitals.

Strain	Secondary hospital	Tertiary hospital		Total
		Provincial	Non-provincial	
CR-KPN	33	102	162	297
CR-ECO	0	11	11	22
CR-ECL	0	4	2	6
CR-KPN/CR-ECO	1	11	7	19
CR-KPN/CR-ECL	0	1	1	2

CR-KPN, carbapenem-resistant *Klebsiella pneumoniae*; CR-ECO, Carbapenem-resistant *Escherichia coli*; CR-ECL, carbapenem-resistant *Enterobacter cloacae*. CR-KPN/CR-ECO samples contain two *species*: CR-KPN and CR-ECO. CR-KPN/CR-ECL samples contain two *species*: CR-KPN and CR-ECL.

Clinical data between patients positive with single infection and mixed infections of CRE were compared (Supplementary Table 3). Multivariable analysis showed that age, cardiovascular diseases were independent risk factors for mixed infections of CRE (Table 4).

**TABLE 4:** Factors independently associated with mixed infections of CRE.

Variables	OR	95% CI	*P*
Age	1.036	1.006–1.067	0.020
Cardiovascular diseases	3.378	1.241–9.174	0.017

Mixed infections of CRE samples contain two *species* CRE: CR-KPN/CR-ECO or CR-KPN/CR-ECL.

Of the 193 positive anal swab samples, KPC, NDM, and both KPC and NDM were found in 150, 9, and 11 strains, respectively. A total of 23 samples showed no presence of tested carbapenemase enzymes.

### Clinical characteristics of the carbapenem-resistant *Enterobacterales* positive and negative patients

According to their CRE test results, the subjects were divided into CRE positive and CRE negative groups, and the clinical data of these two groups was compared (Supplementary Table 1).

### Factors independently associated with carbapenem-resistant *Enterobacterales* positivity

Multivariable analysis showed that length of ICU stay, chronic neurological diseases, transfer from other hospitals, previous infection, and history of antibiotic exposure (carbapenems, beta-lactamase inhibitors, or polymyxins) were independent risk factors for CRE positivity (Table 5).

**TABLE 5:** Factors independently associated with CRE positive.

Variables	OR	95% CI	*P*
Length of ICU stay	1.012	1.003–1.022	0.010
APACHE II score	1.021	1.000–1.043	0.05
Chronic neurological diseases	1.965	1.344–2.874	< 0.001
**Source of patients**			
Newly admitted	Ref		
Transferred from other departments of the same hospital	1.035	0.725–1.479	0.848
Transferred from non-ICU departments of other hospitals	2.114	1.079–4.149	0.029
Transferred from ICU of other hospitals	3.145	1.815–5.434	< 0.001
Previous infection	1.996	1.355–2.941	< 0.001
Carbapenems	2.415	1.642–3.559	< 0.001
Beta-lactamase inhibitors	2.075	1.481–2.907	< 0.001
Polymyxins	25.641	3.155–200	0.002

CRE, Carbapenem-resistant *Enterobacterales*; ICU, intensive care units; OR, odds ratio; CI, confidence interval. There was no multicollinearity between independent variables.

### Carbapenem-resistant *Enterobacterales* positive rates and clinical data in different level hospitals

Of the 78 hospitals, the CRE positive rate was 15.06 and 25.62% in secondary and tertiary hospitals (*P* = 0.004), and 28.43 and 24.02% in provincial and non-provincial hospitals (*P* = 0.164), respectively (Table 6).

**TABLE 6:** CRE positive rates in different level hospitals.

Hospital level	Secondary hospital	Tertiary hospital		Total
		Provincial	Non-provincial	
Positive	25	87	129	241
Negative	141	219	408	768
Positivity rate	15.06%	28.43%	24.02%	23.86%

CRE, Carbapenem-resistant *Enterobacterales*.

According to the hospital levels, the subjects were divided into two groups, including the secondary and tertiary hospital groups, and the baseline characteristics of the two groups were compared. Factors with significant differences between the two groups included body temperature, C-reactive protein (CRP), creatinine, albumin, length of hospital stay, source of patients, previous infection, renal replacement therapy, immunosuppressive agents, non-invasive ventilator, gastroscope, enema, arterial catheter, carbapenems, 3/4 generation cephalosporins, and tigecycline (all *P* < 0.05) (Supplementary Table 2).

## Discussion

There is a growing concern about CRE infection worldwide, which has become a major challenge in the health care. In this multicenter cross-sectional study, we aimed to evaluate the distribution, epidemiological characteristics, and risk factors for CRE positivity in ICUs in Henan Province. Our results suggested that CRE positivity was very common in ICUs. According to the 2018 and 2019 National Antibiotic Resistance Surveillance Reports, CRE infections remain on the rise in China (Committee of Experts on Rational Drug Use of National Health Commission of the P.R.China, 2019, 2020). The overall resistance rate of *Klebsiella pneumoniae* to carbapenems in 2019 was 10.9%, an increase of 0.8% from 2018, and geographical differences were significant, with Henan Province reporting the highest rate (32.8%), which was consistent with our findings (Committee of Experts on Rational Drug Use of National Health Commission of the P.R.China, 2019). Besides, our results the CRE rate were higher than the rates reported by Chen et al. (2017), which may be due to the regional differences (Committee of Experts on Rational Drug Use of National Health Commission of the P.R.China, 2019).

Preventive isolation and active surveillance can help reduce CRE colonization and infection rates (Tucker et al., 2019). Therefore, screening is very important in preventing and controlling CRE. Fecal, rectal swab, and perianal swab cultures are considered the best methods for specimen collection and analysis, but their accuracy decreases gradually. Rectal swabs, however, are recommended as the most suitable clinical specimens in most cases due to their clinical feasibility (Wang et al., 2018). The specimens tested in this study were pharyngeal and anal swabs, and the positive rates of CRE in the pharyngeal and anal swabs were 15.16 and 19.13%, respectively. We found that anal swabs had a higher positive rate than pharyngeal swabs, suggesting that anal swab collection could be a better method for screening for CRE.

Our screening identified CRE strain CP-KPN as the most prevalent, which is consistent with previous reports of the National Antibiotic Resistance Surveillance Program (Committee of Experts on Rational Drug Use of National Health Commission of the P.R.China, 2019, 2020). Moreover, Interestingly, we found that age and cardiovascular diseases were independent risk factors for mixed infections of CRE. Several resistance mechanisms exist in the CRE, including carbapenemase production. KPC, NDM, and OXA-48 were the most common carbapenemases (Iovleva and Doi, 2017). Similar to the findings of Yan et al., we only detected KPC and NDM (Yan et al., 2021). CRE prevalence and carbapenemase types vary greatly by geography. KPC-producing *Enterobacterales* are mainly found in the United States, Colombia, Argentina, Greece, and Italy. In India and Pakistan, MBL NDM-1 is the predominant carbapenemase-producing drug-resistant strain, whereas OXA-48-producing drug-resistant strains are prevalent in Turkey, Sri Lanka, the Middle East, and North Africa (Suay-García and Pérez-Gracia, 2019; Xu et al., 2021). The isolates of CRE in China are predominantly KPC-2, NDM, and OXA-48-producing, with KPC-2 being the most common (Han et al., 2020; Ningjun et al., 2021). In our study, KPC was detected in most positive anal swab samples, which showed similar results to previous studies.

A systematic review of 92 studies showed that the use of antibiotics, previous CRE colonization, mechanical ventilation, previous ICU admission, dialysis, catheterization, length of hospital stay, comorbidities, APACHE II score, and tracheal intubation were the risk factors for CRE infection (Palacios-Baena et al., 2021). In our study, logistic regression analysis determined that length of ICU stay, chronic neurological diseases, transfer from other hospitals, previous infection, and history of antibiotic exposure (carbapenems, beta-lactamase inhibitors, and polymyxins) were independent risk factors for CRE positivity, which was similar to the study by Salomão et al. (2020) except for chronic neurological diseases. Stroke was the most common chronic neurological disorder. Studies have shown that stroke patients hospitalized in the ICU have a high incidence of pulmonary infection and are susceptible to sepsis (Xu et al., 2021). As a result, chronic neurological diseases accompanied by a high incidence of CRE colonization may be explained. The use of antibiotics and tracheal intubation before infection were independent risk factors for CRE infection, which needed increased clinical attention (Shiqi, 2020).

This study showed that the CRE positive rate was significantly higher in tertiary hospitals compared with secondary hospitals in Henan Province. There are several explanations for this, including the higher number of patients transferring from other hospitals or other departments of the same hospital, the administration of invasive procedures, and the exposure to antibiotics. In addition, patients in tertiary hospitals had more severe conditions.

To the best of our knowledge, this was the first study to perform CRE active screening in Henan Province, which involved 1,009 patients from ICUs and 78 hospitals with different levels. However, our study suffered from some limitations. First, regardless of the large number of hospitals involved, and the procedure of sampling was not standardized enough. Second, we only evaluate the prevalence and risk factors of CRE positivity, and the management and follow-up of patients were not reported. Third, our study lacks a genetic component and whole-genome sequencing analysis to support the data. Fourth, our study was only conducted in the Henan Province, and our results cannot be generalized to other areas of China. Last, our study was a cross-sectional study, no conclusions on causality can be drawn. Further large-scale studies with follow-up are warranted to confirm our results.

## Conclusion

In ICUs in Henan Province, CRE positive rate was very high, mostly KPC-type CR-KPN. Patients with prolonged ICU stay, chronic neurological disease, transfer from other hospitals, previous infection, and history of antibiotic exposure are prone to CRE. Age and cardiovascular diseases are susceptibility factors for mixed infections of CRE. The CRE positive rate in tertiary hospitals was higher than that in secondary hospitals, which may be related to the source of patients, antibiotic exposure, disease severity, and previous infection. These findings would provide valuable evidence for anti-infective therapy and infection prevention of CRE positive.

## Data availability statement

The original contributions presented in this study are included in the article/Supplementary material, further inquiries can be directed to the corresponding authors.

## Ethics statement

This study was approved by the medical ethics committee of Henan People’s Hospital, Ethics Number: (2020) Ethical Review No. (143). The patients/participants provided their written informed consent to participate in this study.

## Author contributions

HS, BQ, HZ, and CS designed the study. SW conducted the carbapenem-resistant *Enterobacterales* detection. ZG collected the data and verified the analyses. BG performed the statistical analyses and wrote the manuscript. HS and BQ proofread and reviewed the manuscript. All authors contributed to the article and approved the submitted version.
