# Supplementary material for: Prevalence and risk factors of carbapenem-resistant Enterobacterales positivity by active screening in intensive care units in the Henan Province of China: A multi-center cross-sectional study
Source: Front Microbiol. 2022 Sep 14;13:894341. doi: 10.3389/fmicb.2022.894341 (PMC9521644; doi:10.3389/fmicb.2022.894341)
Supplement: Supplementary file 1 [file Data_Sheet_1.zip › Acknowledgment.docx]

**Acknowledgment**

Anal and pharyngeal swab samples were provided by the following hospitals: Fuwai Huazhong Cardiovascular Hospital, the First Affiliated Hospital of Henan University, Huaihe Hospital of Henan University, First Affiliated Hospital of Henan University of Science and Technology, Yellow River Central Hospital, Henan Provincial People’s Hospital, Henan Provincial Chest Hospital, Henan NO.3 Provincial People’s Hospital, Henan Provincial Staff Hospital, Henan Provincial Hospital of Traditional Chinese Medicine, Henan Cancer Hospital, the First Affiliated Hospital of Henan University of Traditional Chinese Medicine, the Third Affiliated Hospital of Xinxiang Medical College, the Second Affiliated Hospital of Zhengzhou University, the Fifth Affiliated Hospital of Zhengzhou University, the First Affiliated Hospital of Zhengzhou University, Hebi People’s Hospital, Yellow River Sanmenxia Hospital, Jiyuan People’s Hospital, Jiaozuo Second People’s Hospital, Jiaozuo People’s Hospital, Kaifeng People’s Hospital, Kaifeng Central Hospital, Kaifeng Traditional Chinese Medicine Hospital, Luoyang Dong Fang Hospital, Luoyang Central Hospital, Luohe Central Hospital, the Second Affiliated Hospital of Luohe Medical College, Nanshi Hospital of Nanyang, Nanyang First People’s Hospital, Nanyang Central Hospital, Pingdingshan Second People’s Hospital, Pingdingshan First People’s Hospital , Pingmei Shenma Medical Group General Hospital, Anyang District Hospital of Puyang City, Puyang People’s Hospital,Puyang Oilfield General Hospital, Puyang Hospital of Traditional Chinese Medicine, Sanmenxia Central Hospital, Shangqiu First People’s Hospital, Xinxiang Second People’s Hospital, Xinxiang First People’s Hospital, Xinxiang Central Hospital, Xinyang Central Hospital, Xuchang Municipal Hospital, Xuchang People’s Hospital, Xuchang Central Hospital, Zhengzhou People’s Hospital, Zhengzhou Sixth People’s Hospital, Zhengzhou Seventh People’s Hospital, Zhengzhou City Third People’s Hospital, Zhengzhou First People’s Hospital, Zhengzhou Orthopedic Hospital, Zhengzhou Central Hospital, Zhoukou Central Hospital, Zhumadian First People’s Hospital, Zhumadian Central Hospital, Xiangcheng County People’s Hospital, Yongcheng People’s Hospital , Zhoukou First People’s Hospital, Xinzheng People’s Hospital, Jiaxian Traditional Chinese Medicine Hospital, Xinxiang County People’s Hospital, Changyuan People’s Hospital, Bo’ai County People’s Hospital, Yexian People’s Hospital, Yanling County People’s Hospital, Yuzhou People’s Hospital, General Hospital of Yima Coal Industry Group Co., Ltd., Minquan County People’s Hospital, Xincai County People’s Hospital, Gushi County People’s Hospital, Gongyi People’s Hospital, Xinmi Traditional Chinese Medicine Hospital, Ruzhou People’s Hospital, Neihuang County People’s Hospital, Xiuwu County People’s Hospital and Dengfeng People’s Hospital.

We thank Zhao Jingge and Liu Yanhong from Henan People’s Hospital for their help in the statistical analysis.
