# Supplementary material for: Prevalence and risk factors of carbapenem-resistant Enterobacterales positivity by active screening in intensive care units in the Henan Province of China: A multi-center cross-sectional study
Source: Front Microbiol. 2022 Sep 14;13:894341. doi: 10.3389/fmicb.2022.894341 (PMC9521644; doi:10.3389/fmicb.2022.894341)
Supplement: Supplementary file 1 [file Data_Sheet_1.zip › Supplementary Table 2.docx]

**Supplementary Table 2 Clinical data of patients in different level hospitals**

| Variables | Secondary hospital(n=166) | Tertiary hospital(n=843) | Z/χ2/t | P |
| --- | --- | --- | --- | --- |
| Age(years) | 67.00(54.00,74.25) | 64.00(51.00,75.00) | -1.342 | 0.180 |
| Sex | | | |  |
| Male | 103 | 539 | 0.214 | 0.660 |
| Female | 63 | 304 |  |  |
| BMI | 23.67(21.66,25.85) | 23.44(21.24,25.49) | -1.054 | 0.292 |
| Body temperature (℃) | 36.7(36.5,37) | 36.8(36.5,37.2) | -3.479 | 0.001 |
| APACHE II score | 17(12.00,20.75) | 16(11,23) | -0.103 | 0.918 |
| WBC (x10^9^/L) | 10.25(8.10,13.30) | 10.81(8.03,14.4) | -1.057 | 0.290 |
| CRP (mg/L) | 36.85(10,96.83) | 54.20(20.81,118.49) | -3.241 | 0.001 |
| Procalcitonin (ng/ml) | 0.41(0.11,1.37) | 0.42(0.15,1.40) | -0.904 | 0.366 |
| Creatinine (µmol/L) | 57(41.15,81.85) | 64.39(47,99) | -2.694 | 0.007 |
| Albumin (g/L) | 33.81±6.57 | 32.78±7.08 | -2.135 | 0.033 |
| Total bilirubin(µmol/L) | 14.21(9.73,20.73) | 14.2(9.40,23.10) | -1.022 | 0.307 |
| Length of hospital stay (days) | 5(2,11) | 7(3,14) | -3.380 | 0.001 |
| Length of ICU stay (days) | 3(2,8) | 5(2,10) | -1.682 | 0.092 |
| **History of disease** |  |  |  |  |
| Cardiovascular diseases | 73(43.98%) | 393(46.62%) | 0.390 | 0.552 |
| Chronic lung diseases | 16(9.64%) | 110(13.05%) | 1.476 | 0.249 |
| Chronic neurological diseases | 38(22.89%) | 149(17.67%) | 2.500 | 0.126 |
| Chronic kidney diseases | 9(5.42%) | 62(7.35%) | 0.792 | 0.413 |
| Peptic ulcer | 7(4.22%) | 30(3.56%) | 0.170 | 0.821 |
| Chronic liver diseases | 5(3.01%) | 35(4.14%) | 0.473 | 0.530 |
| Diabetes | 28(16.87%) | 169(20.05%) | 0.893 | 0.392 |
| HIV infection | 0 | 4(0.47%) | 0.046 | 0.615 |
| Connective tissue diseases | 0 | 7(0.83%) | 0.444 | 0.375 |
| Blood diseases | 2(1.20%) | 8(0.95%) | 0.093 | 1.000 |
| Malignant tumors | 5(3.01%) | 43(5.10%) | 1.336 | 0.320 |
| Immunosuppressed state | 1(0.60%) | 24(2.85%) | 2.037 | 0.077 |
| **Source of patients** |  |  |  |  |
| Newly admitted | 98(59.04%) | 359(42.59%) | 20.399 | <0.001 |
| Transferred from other departments of the same hospital | 59(35.54%) | 363(43.06%) |  |  |
| Transferred from non-ICU departments of other hospitals | 1(0.60%) | 52(6.17%) |  |  |
| Transferred from ICU of other hospitals | 8(4.82%) | 69(8.19%) |  |  |
| Previous infection | 21(12.65%) | 172(20.40%) | 5.389 | 0.023 |
| Renal replacement therapy | 7(4.22%) | 93(11.03%) | 7.215 | 0.010 |
| Immunosuppressant | 1(0.60%) | 35(4.15%) | 5.078 | 0.035 |
| **Intrusive or invasive procedures** | 132(79.52%) | 694(82.33%) | 0.736 | 0.440 |
| Invasive ventilator | 82(49.40%) | 455(53.97%) | 1.167 | 0.307 |
| Non-invasive ventilator | 3(1.81%) | 50(5.93%) | 4.739 | 0.034 |
| Surgery | 56(33.73%) | 328(38.91%) | 1.575 | 0.222 |
| Gastroscopy | 0 | 30(3.56%) | 4.917 | 0.027 |
| Colonoscopy | 2(1.20%) | 12(1.42%) | 0.048 | 1.000 |
| Enema | 21(12.65%) | 174(20.64%) | 5.679 | 0.018 |
| Central venous catheter | 98(59.04%) | 524(62.16%) | 0.572 | 0.485 |
| Arterial catheter | 15(9.04%) | 328(38.91%) | 55.157 | <0.001 |
| Gastric tube | 110(66.27%) | 512(60.74%) | 1.794 | 0.191 |
| Indwelling catheter | 127(76.51%) | 649(76.99%) | 0.018 | 0.920 |
| **Drainage tube** | | | | |
| No drainage | 113(68.07%) | 557(66.07%) | 9.018 | 0.060 |
| Head | 31(18.67%) | 104(12.34%) |  |  |
| Thoracic cavity | 7(4.22%) | 59(7.00%) |  |  |
| Abdominal cavity | 8(4.82%) | 65(7.71) |  |  |
| Others | 7(4.22%) | 58(6.88%) |  |  |
| **History of antibiotic exposure** | 122(73.49%) | 673(79.83%) | 3.336 | 0.077 |
| Carbapenems | 12(7.23%) | 199(23.63%) | 22.548 | <0.001 |
| Beta-lactamase inhibitors | 63(37.95%) | 323(38.32%) | 0.008 | 0.931 |
| 3/4 generation Cephalosporin | 44(26.51%) | 109(12.93%) | 19.870 | <0.001 |
| Tigecycline | 1(0.60%) | 41(4.86%) | 6.313 | 0.017 |
| Polymyxins | 0(0.00%) | 12(1.42%) | 1.334 | 0.234 |
| Others | 33(19.88%) | 224(26.57%) | 3.272 | 0.079 |

CRE: Carbapenem-resistant *Enterobacterales*; BMI: body mass index; WBC: white blood count; CRP: C-reactive protein; ICU: intensive care units; HIV: human immunodeficiency virus. The APACHE II score were derived from the worst values in the first 24 hours after admission to the ICU, the other parameters were collected upon the day of sampling.
